# Crystal structure of the one-dimensional coordination polymer formed by the macrocyclic [Ni(cyclam)]^2+^ cation and the dianion of di­phenyl­silanediylbis(4-benzoic acid)

**DOI:** 10.1107/S2056989020006544

**Published:** 2020-05-29

**Authors:** Sergey P. Gavrish, Sergiu Shova, Maria Cazacu, Yaroslaw D. Lampeka

**Affiliations:** a L.V. Pisarzhevskii Institute of Physical Chemistry of the National Academy of Sciences of Ukraine, Prospekt Nauki 31, Kyiv 03028, Ukraine; b "Petru Poni" Institute of Macromolecular Chemistry, Department of Inorganic Polymers, Aleea Grigore Ghica Voda 41A, RO-700487 Iasi, Romania

**Keywords:** crystal structure, macrocyclic ligand, cyclam, nickel, coordination polymers, hydrogen bonds

## Abstract

The asymmetric unit of the title compound, *catena*-poly[[[(1,4,8,11-tetra­aza­cyclo­tetra­decane-κ^4^ *N* ^1^,*N* ^4^,*N* ^8^,*N* ^11^)nickel(II)]-μ-4,4′-(di­phenyl­silanedi­yl)dibenz­o­ato-κ^2^ *O*:*O*′] sesquihydrate], {[Ni(C_26_H_18_O_4_Si)(C_10_H_24_N_4_)]·1.5H_2_O}_*n*_, consists of the halves of the centrosymmetric macrocyclic cation and the *C*_2_-symmetric di­carboxyl­ate dianion and of the water mol­ecule of crystallization. The Ni^2+^ ion is coordinated by the four secondary N atoms of the macrocyclic ligand characterized by the most energetically favourable *trans*-III conformation and two mutually *trans* O atoms of the carboxyl­ate, forming a slightly tetra­gonally elongated *trans*-N_4_O_2_ octa­hedron. The crystals are composed of parallel polymeric chains of the macrocyclic cations linked by the anions of the acid running along the [101] direction. Each polymeric chain is bonded to four neighbouring ones *via* water mol­ecules providing O—H⋯O hydrogen bonds to the non-coordinated carboxyl O atoms to form a three-dimensional supra­molecular network.

## Abstract 2

The title coordination polymer consists of parallel zigzag-like chains of [Ni(cyclam)]^2+^ cations bridged by the dianions of the acid in the axial positions of the *trans*-NiN_4_O_2_ coordination polyhedron. Polymeric chains propagating along the [101] direction are assembled into a three-dimensional network by O—H⋯O hydrogen bonds.

## Chemical context   

Aromatic carboxyl­ates are the most popular ligands employed as linkers joining metal-containing fragments (secondary building units, SBUs) in the construction of coordination polymers (Rao *et al.*, 2004 ▸). This class of hybrid organic–inorganic materials possesses great potential for applications in gas storage, separation, catalysis, *etc*. (MacGillivray & Lukehart, 2014 ▸; Kaskel, 2016 ▸). At the same time, carboxyl­ate linkers containing a silicon core are still rare objects of investigation, although it is assumed that the presence of these heteroatoms may affect the topology and properties of the resulting coordination polymers, which are known to be rather sensitive to tiny structural variations in the constituting parts. Besides these structural aspects, carboxyl­ate ligands containing heteroatoms are of current inter­est as precursors for the preparation of structured heteroatom-doped carbonaceous materials possessing excellent electron conductivity, high porosity and diverse applications, including electrocatalysis and energy storage and conversion (Yang *et al.*, 2019 ▸; Zhong *et al.*, 2019 ▸).

Di­phenyl­silanediylbis(4-benzoic acid), a dicarboxylate possessing a characteristic bent shape, has been already utilized for the synthesis of coordination polymers with tetra­nuclear Zn^II^ (Liu *et al.*, 2009 ▸) and dinuclear Zn^II^ and Mn^II^ (Turcan-Trofin *et al.*, 2018 ▸) SBUs, as well as a copper(II) complex with 1,10-phenanthroline as co-ligand (Cazacu *et al.*, 2014 ▸). However, no attempt has been made thus far to combine this linker with macrocyclic complexes, which provide pre-formed SBUs of another type (two vacant *trans* axial positions in the coordination sphere of the metal ion) with an additional benefit of extremely high thermodynamic stability and kinetic inertness (Melson, 1979 ▸; Yatsimirskii & Lampeka, 1985 ▸). At the same time, such SBUs have been used successfully for the assembly of a number of coordination polymers (Lampeka & Tsymbal, 2004 ▸; Suh & Moon, 2007 ▸; Suh *et al.*, 2012 ▸; Stackhouse & Ma, 2018 ▸), including those with some other Si-containing carboxyl­ates (Gavrish *et al.*, 2020*a* ▸; Gavrish *et al.*, 2020*b* ▸).
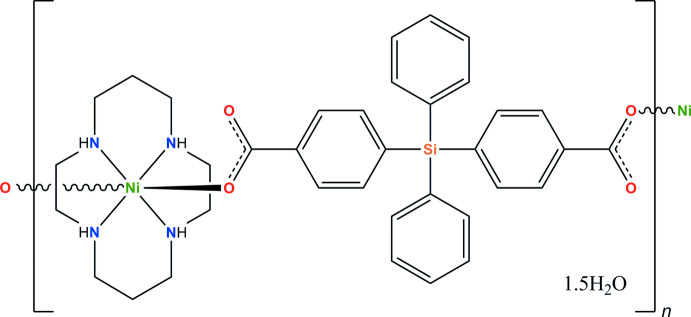


As part of our research on such compounds, we report herein the synthesis and crystal structure of the coordination polymer built up from the nickel(II) complex of the 14-membered macrocyclic ligand 1,4,8,11-tetra­aza­cyclo­tetra­decane (cyclam, *L*), and the dianion of di­phenyl­silane­diylbis(4-benzoic acid) (H_2_A), *viz.*, *catena*-poly[[[(1,4,8,11-tetra­aza­cyclo­tetra­decane-*κ* ^4^ *N* ^1^ *N* ^4^ *N* ^8^ *N* ^11^)nickel(II)]-*μ*-di­phen­yl­silanediylbis(4-benzoato)-*κ* ^2^ *O*:*O′*] sesquihydrate], [Ni(*L*)(A)·1.5H_2_O]_*n*_, (**I**).

## Structural commentary   

**Figure 1:**
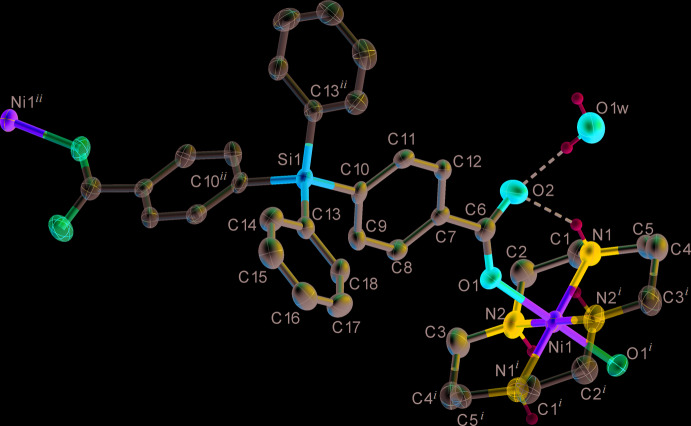
The extended asymmetric unit in **I** showing the coordination environment of the Ni atoms and the atom-labelling scheme (displacement ellipsoids are drawn at the 40% probability level). The atoms obtained by symmetry transformations are shown with 50% transparency. C-bound H atoms are omitted for clarity. Dashed lines represent hydrogen-bonding inter­actions. [Symmetry codes: (i) −*x* + 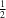, −*y* + 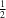, −*z*; (ii) −*x* + 1, *y*, −*z* + 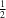].

The mol­ecular structure of the title compound **I** is shown in Fig. 1 ▸. It represents a one-dimensional coordination polymer built of the centrosymmetric macrocyclic [Ni(*L*)]^2+^ cations coordinated in axial positions by the oxygen atoms of the carboxyl groups of the acid.

The macrocyclic ligand in the complex cation adopts the most abundant energetically favourable *trans*-III (*R,R,S,S*) conformation (Bosnich *et al.*, 1965 ▸) with almost equal Ni—N bond lengths (Table 1 ▸). The five-membered chelate rings are present in *gauche* and the six-membered in *chair* conformations. The geometric parameters observed are characteristic of high-spin nickel(II) complexes with 14-membered tetra­amine ligands (Lampeka & Tsymbal, 2004 ▸). The axial Ni—O bond lengths are somewhat longer than the Ni—N ones resulting in a slight tetra­gonal distortion of the *trans*-N_4_O_2_ nickel(II) coordination polyhedron. The location of the metal ion on the inversion centre enforces strict planarity of the equatorial Ni(N_4_) fragment.

**Figure 2:**
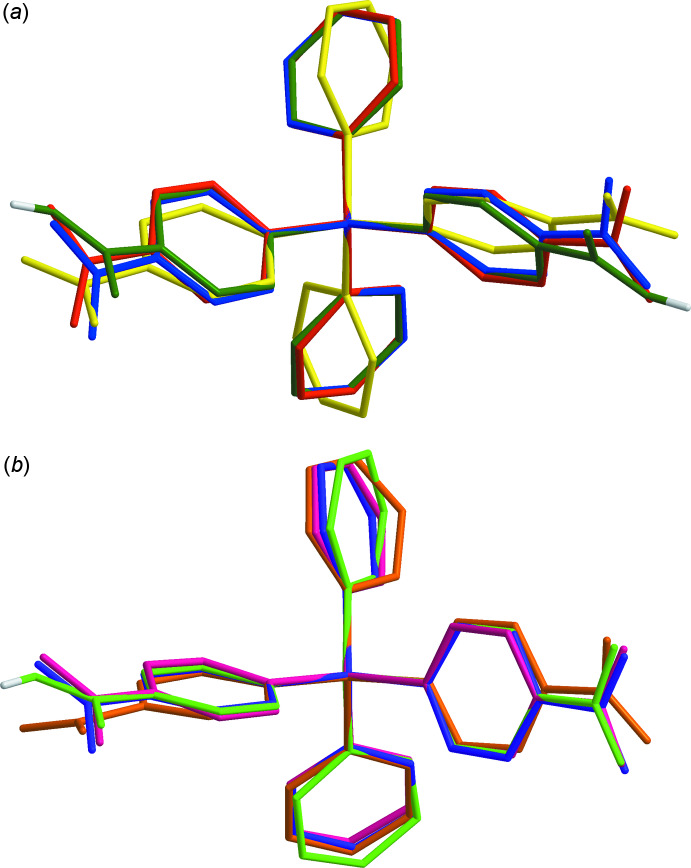
Comparison of the conformations of di­phenyl­silanediylbis(4-benzoic acid) and its anions. (*a*) C_2_-symmetric structures. **H_2_A**: dark-green – XOZVIT; **A^2−^**: red – **I** (current work), blue – ZIGXEV-1, yellow – XOQXIL-1. (*b*) Asymmetric structures. **HA^−^**: light-green – XOZWAM; **A^2−^**: lilac – ZIGXIZ-1, pink – ZIGXIZ-2, orange – XOQXIL-2. Numbers accompanying refcodes refer to two structurally non-equivalent anions in a given compound. The disordered symmetric anion in ZIGXEV-2 is not shown.

The dianion of the acid in complex **I** possesses intrinsic twofold axial symmetry, with the Si atom lying on the rotation axis. An analogous *C*_2_-symmetric conformation was found [Cambridge Structural Database (CSD, Version 5.40, last update February 2019; Groom *et al.*, 2016 ▸)] for the mol­ecules/anions of the acid in the structures XOZVIT (Cazacu *et al.*, 2014 ▸) and ZIGXEV (Turcan-Trofin *et al.*, 2018 ▸). In two cases [XOZWAM (Cazacu *et al.*, 2014 ▸) and ZIGXIZ (Turcan-Trofin *et al.*, 2018 ▸)], the carboxyl­ate is present in an asymmetric conformation. At the same time, the coordination polymer XOQXIL (Liu *et al.*, 2009 ▸) includes dianions of the acid in both *C*_2_-symmetric and asymmetric conformations. All these data are summarized in Fig. 2 ▸, which clearly illustrates the capability of rotation of aromatic rings in the tetra­phenyl­silane moiety around the Si—C_ar­yl_ bonds by a wide range of angles. Another feature worth noting is that the symmetric and asymmetric species in fact refer to essentially different types with minor structural variations within each group, with the exception of anion XOQXIL-1.

The carboxyl groups in **I** are coordinated in a monodentate fashion *via* the O1 atom. The non-coordinated O2 atom is involved as proton acceptor in strong hydrogen bonding with the NH group of the macrocycle (Fig. 1 ▸, Table 2 ▸), a situation that is frequently observed in carboxyl­ate complexes of cyclam-like ligands. Almost identical C—O bond lengths [C6—O1 = 1.254 (4) and C6—O2 = 1.260 (4) Å] support the model of essential electronic delocalization in the carboxyl­ate group. The carboxyl group is tilted with respect to the plane of benzene ring by 23.6 (2)°. In general, this angle is prone to large variations, *e.g.* for the structures presented in Fig. 2 ▸ it spans the range 4.1–30.1°.

## Supra­molecular features   

**Figure 3:**
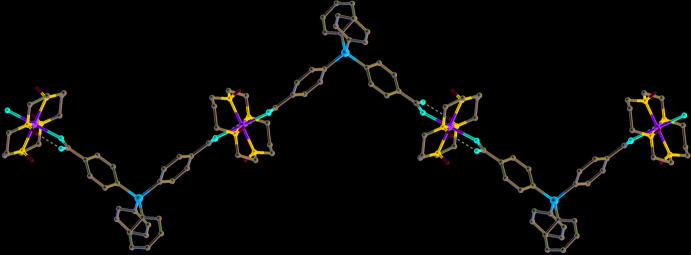
The structure of the polymeric chain in **I**. C-bound H atoms are omitted for clarity.

The crystals of **I** are composed of polymeric chains of [Ni(*L*)]^2+^ cations bridged by the carboxyl­ate ligands, which propagate along the [101] direction. These chains have a distinctive zigzag shape with a chain link length (Si⋯Si distance) of 17.854 (3) Å and an almost ideal tetra­hedral angle (Si⋯Si⋯Si) of 109.09 (2)°(Fig. 3 ▸). The nickel(II) cations in a chain are arranged in line with an Ni⋯Ni separation of 14.543 (2) Å.

**Figure 4:**
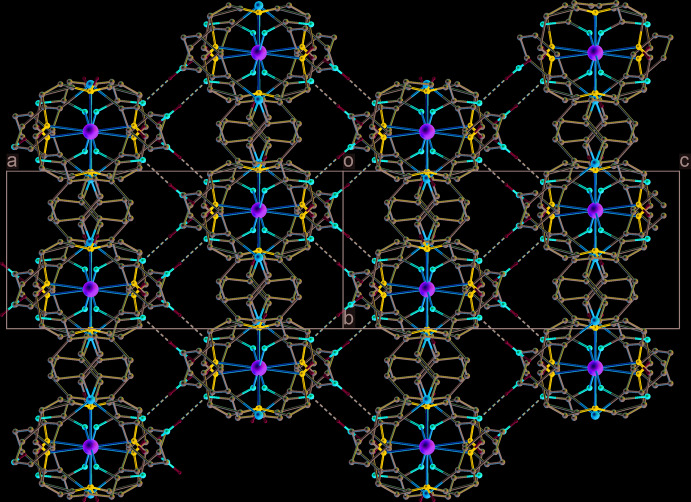
The packing in **I** viewed down the [101] direction with polymeric chains cross-linked by O2⋯H—O1*W*—H⋯O2 hydrogen bonds (dotted lines) to form a three-dimensional supra­molecular network. C-bound H atoms are omitted for clarity.

In the crystal, each such chain is linked to four neighbouring ones due to formation of water-mediated hydrogen bonds between the non-coordinated O2 atoms of the carboxyl group: O2⋯HO1*W*—H⋯O2 (Table 2 ▸). In turn, each O2 atom is involved in hydrogen bonding with two H_2_O mol­ecules [symmetry codes: *x*, *y*, *z*; −*x* + 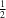, *y* − 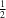, −*z* + 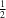], which in conjunction with the N1—H⋯O2 hydrogen bond makes it a triple proton acceptor, while the water mol­ecule serves as a proton donor only. Thus, the water mol­ecules of crystallization play a key role in assembling the one-dimensional polymeric chains into a three-dimensional supra­molecular network (Fig. 4 ▸).

## Synthesis and crystallization   

All chemicals and solvents used in this work were purchased from Sigma–Aldrich and were used without further purification. The macrocyclic nickel(II) complex Ni(*L*)(ClO_4_)_2_ (Barefield *et al.*, 1976 ▸) and di­phenyl­silanediylbis(4-benzoic acid) (Cazacu *et al.*, 2014 ▸) were prepared according to procedures described previously.

**{Ni(** ***L*** **)(A)·1.5H_2_O}** ***_n_***, (**I**). A solution of 100 mg (0.236 mmol) of the acid (H_2_A) in 24.5 ml of DMF containing 0.3 ml of tri­ethyl­amine was thoroughly layered on top of the solution of 120 mg (0.262 mmol) of [Ni(*L*)](ClO_4_)_2_ in 7.5 ml of water and the tightly closed system was left for two weeks at room temperature. The crystalline precipitate formed was filtered off, washed with DMF, methanol and dried in air. Yield 157 mg (94%). Analysis calculated for C_72_H_90_N_8_Ni_2_O_11_Si_2_: C, 61.03; H, 6.40; N, 7.91%. Found: C, 61.16; H, 6.36; N, 8.09%.

Single crystals of **I** in the form of light-yellow prisms suitable for X-ray diffraction analysis were obtained analogously using *ca* 10 times lower concentration of reagents.

**Safety note**: Perchlorate salts of metal complexes are potentially explosive and should be handled with care.

## Refinement   

Crystal data, data collection and structure refinement details are summarized in Table 3 ▸. All H atoms were placed in geometrically idealized positions and constrained to ride on their parent atoms, with C—H distances of 0.93 (ring H atoms) or 0.97 Å (open-chain H atoms), an N—H distance of 0.98 Å and an aqua O—H distance of 0.85 Å with *U*_iso_(H) values of 1.2 or 1.5*U*_eq_ times that of the parent atoms. Since the water mol­ecule of crystallization at full occupancy exhibited unreasonably high displacement ellipsoids, its occupancy parameter was reduced to 75%.

## Supplementary Material

Crystal structure: contains datablock(s) I. DOI: 10.1107/S2056989020006544/hb7914sup1.cif

Structure factors: contains datablock(s) I. DOI: 10.1107/S2056989020006544/hb7914Isup2.hkl

CCDC reference: 2004084

Additional supporting information:  crystallographic information; 3D view; checkCIF report

## Figures and Tables

**Table 1:** Selected bond lengths and angles (Å, °)

Distances		Bite angles	
Ni1—N1	2.066 (3)	N1—Ni1—N2	86.05 (13)
Ni1—N2	2.068 (3)	N1—Ni1—N2^i^	93.95 (13)
Ni1—O1	2.128 (2)		

Symmetry code: (i) −*x* + 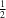, −*y* + 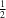, −*z*.

**Table 2:** Hydrogen-bond geometry (Å, °)

*D*—H⋯*A*	*D*—H	H⋯*A*	*D*⋯*A*	*D*—H⋯*A*
N1—H1⋯O2	0.98	2.02	2.916 (4)	151
O1*W*—H1*WA*⋯O2^i^	0.85	2.05	2.850 (5)	156
O1*W*—H1*WB*⋯O2	0.85	1.89	2.744 (5)	177

Symmetry code: (i) 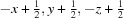.

**Table 3:** Experimental details

Crystal data	
Chemical formula	[Ni(C_26_H_18_O_4_Si)(C_10_H_24_N_4_)]·1.5H_2_O
*M*_r_	1417.11
Crystal system, space group	Monoclinic, *C*2/*c*
Temperature (K)	200
*a*, *b*, *c* (Å)	25.390 (4), 7.3865 (10), 18.2424 (16)
β (°)	98.161 (10)
*V* (Å^3^)	3386.6 (8)
*Z*	2
Radiation type	Mo *K*α
μ (mm^−1^)	0.66
Crystal size (mm)	0.35 × 0.10 × 0.10

Data collection	
Diffractometer	Agilent Xcalibur, Eos
Absorption correction	Multi-scan (*CrysAlis PRO*; Agilent, 2014 ▸),
*T*_min_, *T*_max_	0.964, 1.000
No. of measured, independent and observed [*I* > 2σ(*I*)] reflections	7999, 3926, 2512
*R*_int_	0.052
(sin θ/λ)_max_ (Å^−1^)	0.693

Refinement	
*R*[*F* ^2^ > 2σ(*F* ^2^)], *wR*(*F* ^2^), *S*	0.066, 0.147, 1.03
No. of reflections	3926
No. of parameters	219
H-atom treatment	H-atom parameters constrained
Δρ_max_, Δρ_min_ (e Å^−3^)	0.51, −0.51

Computer programs: *CrysAlis PRO* (Agilent, 2014 ▸), *SHELXT* (Sheldrick, 2015*a* ▸), *SHELXL2018/3* (Sheldrick, 2015*b* ▸), *OLEX2* (Dolomanov *et al.*, 2009 ▸) and *publCIF* (Westrip, 2010 ▸).

## supplementary crystallographic information

### Crystal data

**Table:**

[Ni(C_26_H_18_O_4_Si)(C_10_H_24_N_4_)]·1.5H_2_O	*F*(000) = 1500
*M**_r_* = 1417.11	*D*_x_ = 1.390 Mg m^−^^3^
Monoclinic, *C*2/*c*	Mo *K*α radiation, λ = 0.71073 Å
*a* = 25.390 (4) Å	Cell parameters from 1577 reflections
*b* = 7.3865 (10) Å	θ = 2.6–29.5°
*c* = 18.2424 (16) Å	µ = 0.66 mm^−^^1^
β = 98.161 (10)°	*T* = 200 K
*V* = 3386.6 (8) Å^3^	Prism, clear light yellow
*Z* = 2	0.35 × 0.10 × 0.10 mm

### Data collection

**Table:**

Agilent Xcalibur, Eos diffractometer	3926 independent reflections
Radiation source: Enhance (Mo) X-ray Source	2512 reflections with *I* > 2σ(*I*)
Graphite monochromator	*R*_int_ = 0.052
Detector resolution: 16.1593 pixels mm^-1^	θ_max_ = 29.5°, θ_min_ = 2.6°
ω scans	*h* = −31→21
Absorption correction: multi-scan (CrysAlisPro; Agilent, 2014),	*k* = −10→9
*T*_min_ = 0.964, *T*_max_ = 1.000	*l* = −21→23
7999 measured reflections	

### Refinement

**Table:**

Refinement on *F*^2^	Primary atom site location: structure-invariant direct methods
Least-squares matrix: full	Hydrogen site location: mixed
*R*[*F*^2^ > 2σ(*F*^2^)] = 0.066	H-atom parameters constrained
*wR*(*F*^2^) = 0.147	*w* = 1/[σ^2^(*F*_o_^2^) + (0.0427*P*)^2^ + 4.2041*P*] where *P* = (*F*_o_^2^ + 2*F*_c_^2^)/3
*S* = 1.03	(Δ/σ)_max_ < 0.001
3926 reflections	Δρ_max_ = 0.51 e Å^−^^3^
219 parameters	Δρ_min_ = −0.51 e Å^−^^3^
0 restraints	

### Special details

**Table:**

Geometry. All esds (except the esd in the dihedral angle between two l.s. planes) are estimated using the full covariance matrix. The cell esds are taken into account individually in the estimation of esds in distances, angles and torsion angles; correlations between esds in cell parameters are only used when they are defined by crystal symmetry. An approximate (isotropic) treatment of cell esds is used for estimating esds involving l.s. planes.

### Fractional atomic coordinates and isotropic or equivalent isotropic displacement parameters (Å^2^)

**Table:**

	*x*	*y*	*z*	*U*_iso_*/*U*_eq_	Occ. (<1)
Ni1	0.250000	0.250000	0.000000	0.0273 (2)	
Si1	0.500000	0.95105 (19)	0.250000	0.0256 (3)	
O1	0.30989 (10)	0.3756 (4)	0.07669 (14)	0.0372 (7)	
O2	0.27252 (11)	0.4301 (4)	0.17836 (15)	0.0514 (8)	
N1	0.19897 (12)	0.2120 (5)	0.07732 (17)	0.0377 (8)	
H1	0.214580	0.275668	0.122344	0.045*	
N2	0.27921 (12)	−0.0047 (4)	0.03013 (18)	0.0381 (8)	
H2	0.262042	−0.087426	−0.007954	0.046*	
C1	0.20090 (17)	0.0146 (6)	0.0952 (2)	0.0464 (11)	
H1A	0.177654	−0.051687	0.057721	0.056*	
H1B	0.188941	−0.005714	0.142697	0.056*	
C2	0.25790 (17)	−0.0508 (6)	0.0976 (2)	0.0480 (11)	
H2A	0.279912	0.003615	0.139702	0.058*	
H2B	0.259044	−0.181115	0.104319	0.058*	
C3	0.33655 (17)	−0.0306 (6)	0.0316 (2)	0.0490 (12)	
H3A	0.345186	−0.156556	0.042523	0.059*	
H3B	0.355364	0.042076	0.071235	0.059*	
C4	0.14439 (17)	0.4804 (6)	0.0397 (2)	0.0477 (11)	
H4A	0.108246	0.524309	0.038067	0.057*	
H4B	0.166204	0.543620	0.079585	0.057*	
C5	0.14505 (15)	0.2795 (6)	0.0585 (2)	0.0435 (11)	
H5A	0.125675	0.260021	0.100024	0.052*	
H5B	0.127171	0.212343	0.016526	0.052*	
C6	0.30912 (15)	0.4468 (5)	0.1389 (2)	0.0319 (9)	
C7	0.35609 (14)	0.5657 (5)	0.16868 (18)	0.0247 (8)	
C8	0.40442 (14)	0.5475 (5)	0.14241 (19)	0.0312 (9)	
H8	0.408530	0.458487	0.107630	0.037*	
C9	0.44678 (14)	0.6610 (5)	0.1675 (2)	0.0329 (9)	
H9	0.479200	0.644164	0.150332	0.039*	
C10	0.44174 (14)	0.8002 (5)	0.21810 (18)	0.0264 (8)	
C11	0.39244 (14)	0.8183 (5)	0.24315 (18)	0.0296 (8)	
H11	0.387380	0.911020	0.275947	0.035*	
C12	0.35091 (14)	0.7001 (5)	0.21976 (19)	0.0309 (9)	
H12	0.319013	0.711608	0.238874	0.037*	
C13	0.51508 (14)	1.1037 (5)	0.1735 (2)	0.0285 (8)	
C14	0.55319 (15)	1.2399 (5)	0.1896 (2)	0.0371 (9)	
H14	0.571043	1.249604	0.237641	0.044*	
C15	0.56512 (17)	1.3597 (6)	0.1370 (2)	0.0466 (11)	
H15	0.590586	1.449113	0.149517	0.056*	
C16	0.53943 (17)	1.3472 (6)	0.0658 (2)	0.0458 (11)	
H16	0.547953	1.426763	0.029713	0.055*	
C17	0.50098 (17)	1.2169 (6)	0.0477 (2)	0.0464 (11)	
H17	0.483033	1.210014	−0.000366	0.056*	
C18	0.48907 (15)	1.0964 (5)	0.1009 (2)	0.0361 (9)	
H18	0.463175	1.008505	0.088027	0.043*	
O1W	0.19429 (16)	0.6528 (6)	0.2165 (2)	0.0640 (12)	0.75
H1WA	0.205149	0.708674	0.256534	0.096*	0.75
H1WB	0.218579	0.582004	0.206074	0.096*	0.75

### Atomic displacement parameters (Å^2^)

**Table:**

	*U*^11^	*U*^22^	*U*^33^	*U*^12^	*U*^13^	*U*^23^
Ni1	0.0236 (4)	0.0255 (4)	0.0312 (4)	−0.0020 (3)	−0.0015 (3)	−0.0022 (3)
Si1	0.0214 (7)	0.0242 (7)	0.0295 (7)	0.000	−0.0019 (6)	0.000
O1	0.0313 (15)	0.0442 (17)	0.0345 (14)	−0.0102 (13)	−0.0009 (11)	−0.0133 (14)
O2	0.0425 (19)	0.065 (2)	0.0492 (17)	−0.0234 (16)	0.0151 (14)	−0.0184 (16)
N1	0.0339 (19)	0.043 (2)	0.0352 (18)	−0.0042 (16)	0.0013 (14)	−0.0049 (16)
N2	0.035 (2)	0.0298 (18)	0.047 (2)	0.0013 (15)	−0.0039 (15)	0.0018 (17)
C1	0.046 (3)	0.039 (2)	0.054 (3)	−0.011 (2)	0.005 (2)	0.004 (2)
C2	0.052 (3)	0.041 (3)	0.051 (3)	0.008 (2)	0.006 (2)	0.016 (2)
C3	0.040 (3)	0.046 (3)	0.060 (3)	0.016 (2)	0.003 (2)	0.002 (2)
C4	0.037 (3)	0.050 (3)	0.053 (3)	0.004 (2)	−0.001 (2)	−0.004 (2)
C5	0.028 (2)	0.059 (3)	0.044 (2)	−0.002 (2)	0.0067 (18)	−0.009 (2)
C6	0.032 (2)	0.025 (2)	0.036 (2)	−0.0021 (17)	−0.0012 (17)	0.0018 (18)
C7	0.0252 (19)	0.0228 (18)	0.0245 (18)	−0.0011 (15)	−0.0023 (14)	0.0033 (16)
C8	0.033 (2)	0.028 (2)	0.031 (2)	0.0018 (17)	−0.0005 (16)	−0.0102 (17)
C9	0.021 (2)	0.033 (2)	0.043 (2)	−0.0005 (17)	0.0023 (16)	−0.0034 (19)
C10	0.0216 (19)	0.0271 (19)	0.0277 (18)	0.0005 (15)	−0.0060 (14)	0.0048 (16)
C11	0.030 (2)	0.034 (2)	0.0238 (18)	−0.0023 (17)	−0.0003 (15)	−0.0045 (17)
C12	0.0223 (19)	0.039 (2)	0.0311 (19)	−0.0028 (17)	0.0026 (15)	−0.0015 (18)
C13	0.024 (2)	0.0255 (19)	0.036 (2)	0.0017 (16)	0.0037 (15)	0.0035 (18)
C14	0.038 (2)	0.032 (2)	0.040 (2)	−0.0025 (19)	0.0004 (17)	0.002 (2)
C15	0.041 (3)	0.032 (2)	0.067 (3)	−0.005 (2)	0.008 (2)	0.004 (2)
C16	0.046 (3)	0.039 (3)	0.054 (3)	0.011 (2)	0.015 (2)	0.017 (2)
C17	0.049 (3)	0.053 (3)	0.036 (2)	0.010 (2)	0.0020 (19)	0.008 (2)
C18	0.028 (2)	0.038 (2)	0.040 (2)	−0.0006 (18)	−0.0031 (17)	0.003 (2)
O1W	0.050 (3)	0.070 (3)	0.072 (3)	0.000 (2)	0.009 (2)	−0.012 (3)

### Geometric parameters (Å, º)

**Table:**

Ni1—O1^i^	2.128 (2)	C4—C5	1.523 (6)
Ni1—O1	2.128 (2)	C5—H5A	0.9700
Ni1—N1	2.066 (3)	C5—H5B	0.9700
Ni1—N1^i^	2.065 (3)	C6—C7	1.518 (5)
Ni1—N2^i^	2.068 (3)	C7—C8	1.386 (5)
Ni1—N2	2.068 (3)	C7—C12	1.381 (5)
Si1—C10^ii^	1.878 (4)	C8—H8	0.9300
Si1—C10	1.878 (4)	C8—C9	1.389 (5)
Si1—C13^ii^	1.875 (4)	C9—H9	0.9300
Si1—C13	1.875 (4)	C9—C10	1.399 (5)
O1—C6	1.254 (4)	C10—C11	1.399 (5)
O2—C6	1.260 (4)	C11—H11	0.9300
N1—H1	0.9800	C11—C12	1.389 (5)
N1—C1	1.493 (5)	C12—H12	0.9300
N1—C5	1.452 (5)	C13—C14	1.398 (5)
N2—H2	0.9800	C13—C18	1.395 (5)
N2—C2	1.455 (5)	C14—H14	0.9300
N2—C3	1.465 (5)	C14—C15	1.371 (5)
C1—H1A	0.9700	C15—H15	0.9300
C1—H1B	0.9700	C15—C16	1.372 (6)
C1—C2	1.520 (5)	C16—H16	0.9300
C2—H2A	0.9700	C16—C17	1.377 (6)
C2—H2B	0.9700	C17—H17	0.9300
C3—H3A	0.9700	C17—C18	1.382 (5)
C3—H3B	0.9700	C18—H18	0.9300
C3—C4^i^	1.497 (6)	O1W—H1WA	0.8499
C4—H4A	0.9700	O1W—H1WB	0.8504
C4—H4B	0.9700		
			
O1—Ni1—O1^i^	180.0	C3^i^—C4—H4A	108.2
N1^i^—Ni1—O1^i^	93.98 (11)	C3^i^—C4—H4B	108.2
N1^i^—Ni1—O1	86.02 (11)	C3^i^—C4—C5	116.3 (4)
N1—Ni1—O1	93.98 (11)	H4A—C4—H4B	107.4
N1—Ni1—O1^i^	86.02 (11)	C5—C4—H4A	108.2
N1^i^—Ni1—N1	180.0	C5—C4—H4B	108.2
N1—Ni1—N2^i^	93.95 (13)	N1—C5—C4	111.6 (3)
N1—Ni1—N2	86.05 (13)	N1—C5—H5A	109.3
N1^i^—Ni1—N2	93.95 (13)	N1—C5—H5B	109.3
N1^i^—Ni1—N2^i^	86.05 (13)	C4—C5—H5A	109.3
N2—Ni1—O1^i^	88.51 (11)	C4—C5—H5B	109.3
N2—Ni1—O1	91.49 (11)	H5A—C5—H5B	108.0
N2^i^—Ni1—O1	88.51 (11)	O1—C6—O2	125.7 (3)
N2^i^—Ni1—O1^i^	91.49 (11)	O1—C6—C7	117.0 (3)
N2—Ni1—N2^i^	180.0	O2—C6—C7	117.3 (3)
C10—Si1—C10^ii^	107.2 (2)	C8—C7—C6	120.6 (3)
C13^ii^—Si1—C10^ii^	111.20 (15)	C12—C7—C6	120.8 (3)
C13—Si1—C10	111.20 (15)	C12—C7—C8	118.5 (3)
C13^ii^—Si1—C10	110.63 (16)	C7—C8—H8	119.7
C13—Si1—C10^ii^	110.63 (16)	C7—C8—C9	120.7 (3)
C13—Si1—C13^ii^	106.1 (2)	C9—C8—H8	119.7
C6—O1—Ni1	132.6 (2)	C8—C9—H9	119.3
Ni1—N1—H1	106.8	C8—C9—C10	121.4 (3)
C1—N1—Ni1	106.1 (2)	C10—C9—H9	119.3
C1—N1—H1	106.8	C9—C10—Si1	119.9 (3)
C5—N1—Ni1	117.0 (3)	C11—C10—Si1	123.0 (3)
C5—N1—H1	106.8	C11—C10—C9	117.1 (3)
C5—N1—C1	112.8 (3)	C10—C11—H11	119.5
Ni1—N2—H2	105.8	C12—C11—C10	121.0 (3)
C2—N2—Ni1	106.0 (2)	C12—C11—H11	119.5
C2—N2—H2	105.8	C7—C12—C11	121.2 (3)
C2—N2—C3	116.2 (3)	C7—C12—H12	119.4
C3—N2—Ni1	116.2 (3)	C11—C12—H12	119.4
C3—N2—H2	105.8	C14—C13—Si1	119.0 (3)
N1—C1—H1A	110.0	C18—C13—Si1	124.3 (3)
N1—C1—H1B	110.0	C18—C13—C14	116.6 (3)
N1—C1—C2	108.6 (3)	C13—C14—H14	118.9
H1A—C1—H1B	108.4	C15—C14—C13	122.2 (4)
C2—C1—H1A	110.0	C15—C14—H14	118.9
C2—C1—H1B	110.0	C14—C15—H15	120.1
N2—C2—C1	111.6 (3)	C14—C15—C16	119.8 (4)
N2—C2—H2A	109.3	C16—C15—H15	120.1
N2—C2—H2B	109.3	C15—C16—H16	120.0
C1—C2—H2A	109.3	C15—C16—C17	120.0 (4)
C1—C2—H2B	109.3	C17—C16—H16	120.0
H2A—C2—H2B	108.0	C16—C17—H17	120.0
N2—C3—H3A	108.9	C16—C17—C18	120.0 (4)
N2—C3—H3B	108.9	C18—C17—H17	120.0
N2—C3—C4^i^	113.2 (3)	C13—C18—H18	119.3
H3A—C3—H3B	107.7	C17—C18—C13	121.4 (4)
C4^i^—C3—H3A	108.9	C17—C18—H18	119.3
C4^i^—C3—H3B	108.9	H1WA—O1W—H1WB	109.5
			
Ni1—O1—C6—O2	14.6 (6)	C8—C9—C10—Si1	−179.6 (3)
Ni1—O1—C6—C7	−164.5 (2)	C8—C9—C10—C11	0.9 (5)
Ni1—N1—C1—C2	38.6 (4)	C9—C10—C11—C12	1.4 (5)
Ni1—N1—C5—C4	−55.8 (4)	C10^ii^—Si1—C10—C9	−51.0 (3)
Ni1—N2—C2—C1	38.2 (4)	C10^ii^—Si1—C10—C11	128.4 (3)
Ni1—N2—C3—C4^i^	−54.2 (4)	C10^ii^—Si1—C13—C14	−68.5 (3)
Si1—C10—C11—C12	−178.0 (3)	C10—Si1—C13—C14	172.6 (3)
Si1—C13—C14—C15	−178.1 (3)	C10^ii^—Si1—C13—C18	114.3 (3)
Si1—C13—C18—C17	177.9 (3)	C10—Si1—C13—C18	−4.6 (4)
O1—C6—C7—C8	−21.4 (5)	C10—C11—C12—C7	−2.8 (5)
O1—C6—C7—C12	155.4 (3)	C12—C7—C8—C9	0.6 (5)
O2—C6—C7—C8	159.4 (4)	C13^ii^—Si1—C10—C9	−172.4 (3)
O2—C6—C7—C12	−23.9 (5)	C13—Si1—C10—C9	70.0 (3)
N1—C1—C2—N2	−53.4 (4)	C13^ii^—Si1—C10—C11	7.0 (3)
C1—N1—C5—C4	−179.3 (3)	C13—Si1—C10—C11	−110.6 (3)
C2—N2—C3—C4^i^	180.0 (4)	C13^ii^—Si1—C13—C14	52.2 (3)
C3—N2—C2—C1	169.0 (3)	C13^ii^—Si1—C13—C18	−125.0 (4)
C3^i^—C4—C5—N1	70.1 (5)	C13—C14—C15—C16	−0.3 (6)
C5—N1—C1—C2	168.0 (3)	C14—C13—C18—C17	0.6 (6)
C6—C7—C8—C9	177.4 (3)	C14—C15—C16—C17	1.3 (6)
C6—C7—C12—C11	−175.1 (3)	C15—C16—C17—C18	−1.3 (6)
C7—C8—C9—C10	−2.0 (5)	C16—C17—C18—C13	0.3 (6)
C8—C7—C12—C11	1.8 (5)	C18—C13—C14—C15	−0.7 (6)

Symmetry codes: (i) −*x*+1/2, −*y*+1/2, −*z*; (ii) −*x*+1, *y*, −*z*+1/2.

### Hydrogen-bond geometry (Å, º)

**Table:**

*D*—H···*A*	*D*—H	H···*A*	*D*···*A*	*D*—H···*A*
N1—H1···O2	0.98	2.02	2.916 (4)	151
O1*W*—H1*WA*···O2^iii^	0.85	2.05	2.850 (5)	156
O1*W*—H1*WB*···O2	0.85	1.89	2.744 (5)	177

Symmetry code: (iii) −*x*+1/2, *y*+1/2, −*z*+1/2.
